# Implementation and Mental Health Outcomes of a Service Cascade Linking Child Welfare and Children’s Mental Health Systems: A Case Study of the Gateway CALL Demonstration

**DOI:** 10.1007/s10488-022-01238-7

**Published:** 2022-11-30

**Authors:** Alicia C. Bunger, Susan Yoon, Kathryn Maguire-Jack, Rebecca Phillips, Kristopher Y. West, Gretchen Clark-Hammond, Christiana Kranich

**Affiliations:** 1 College of Social Work, The Ohio State University, Columbus, OH 43210 USA; 2 School of Social Work, University of Michigan, Ann Arbor, MI 48109 USA; 3 Nationwide Children’s Hospital, Columbus, OH 43205 USA; 4 Mighty Crow Media, LLC, Worthington, OH 43214 USA; 5 Government Resource Center, Ohio Colleges of Medicine, Columbus, OH 43210 USA

**Keywords:** Children’s mental health services, Child welfare, Implementation, Access, Service cascade

## Abstract

The mental health needs of children and youth involved in the child welfare system remain largely unmet. Service cascades are an emerging approach to systematizing mental health screening, assessment, and treatment referral processes. However, evidence is minimal and inconsistent regarding the effectiveness of such approaches for improving mental health service access and outcomes. In an effort to address this gap, this study presents a case-study of the implementation fidelity and treatment outcomes of the Gateway CALL service cascade. Study analyses involved longitudinal data collected as part of a larger evaluation of Gateway CALL. Specifically, descriptive and linear mixed model analyses were conducted to assess the implementation of service cascade components, and changes in mental health outcomes (behavior problems) among 175 children placed out-of-home during the study. Study analyses found that although fidelity was strong early in the service cascade, implementation began to break down once components involved more than one service system (child welfare, mental health). However, results also indicated that parent-reported child behavior problems decreased significantly over time, despite later cascade components being implemented with poor fidelity to the Gateway CALL service model. For children and youth involved in child welfare systems, service cascades like Gateway CALL have the potential to significantly improve both mental health service receipt and outcomes. To maximize the effectiveness of such approaches, later phases of implementation may require increased attention and support, particularly regarding processes and outcomes that cross child welfare and mental health service systems.

**Supplementary Information:**

The online version contains supplementary material available at 10.1007/s10488-022-01238-7.

Children and youth involved in the child welfare system have extensive mental health service needs that often remain unmet (Horwitz et al., 2012; Stein et al., 2016). *Service cascades* involve systematic screening and assessment in one system and referral to treatment in another (e.g., Belenko et al., 2017). When implemented in youth-serving systems, these interventions have potential to improve children’s access to mental health care, and ultimately, their well-being. However, implementing service cascades requires the introduction and alignment of multiple components across multiple organizations. The challenges of implementing service cascades with fidelity have been well-described (Akin et al., 2017; Van Deinse et al., 2019) and may explain, in part, mixed evidence of their effectiveness for improving treatment access and outcomes for children in child welfare (Bunger et al., 2021; Pullmann et al., 2018). This study examines the implementation fidelity of Gateway CALL (Consultation, Assessment, Linkage, and Liaison), a service cascade designed to improve access to mental health treatment for children in out-of-home placements and the effect of service receipt on children’s mental health.

## Linking Child Welfare and Mental Health Systems to Address Unmet Service Needs

Experiencing abuse, neglect, and other traumas as a child can lead to emotional and behavioral problems (Garcia et al., 2017; Kisiel et al., 2017; Yoon et al., 2017; Zhang & Mersky, 2020). As a result, mental health problems are prevalent among children and youth involved in the child welfare system. Approximately 49% of all system-involved children have mental health service needs for various mental disorders including Attention-Deficit/Hyperactivity Disorder, conduct disorder, oppositional defiant disorder, anxiety, depressive disorders, and post-traumatic stress disorder (Bronsard et al., 2016). These rates are particularly high among children who enter out-of-home placements, such as foster care (Engler et al., 2022; Turney & Wildeman, 2016). For example, one study examined mental health problems among adolescents in the child welfare system and found that youth with prior out-of-home placement were 2.29 times more likely to report a mental health problem compared to those with no history of out-of-home placement (Heneghan et al., 2013). Despite high rates of mental health service needs, only about half of child welfare system-involved children receive mental health services (Horwitz et al., 2012; Stein et al., 2016), and even fewer receive care consistent with national standards for screening, assessment, and referral to treatment (Raghavan et al., 2010).

Contact with child welfare workers can serve as a gateway to mental health treatment (Leslie et al., 2005), and as a result, children and youth who enter foster care or other out-of-home placements are often more likely to receive mental health treatment than children who remain at home (Horwitz et al., 2012; Hurlburt et al., 2004; Kim et al., 2021; Raghavan et al., 2010). Although many foster care placement organizations (private organizations that recruit foster parents and place children in foster homes) deliver mental health services as part of a diverse set of case management and other support services (Chuang et al., 2014), children in out-of-home care have substantial unmet mental health service needs (Turney & Wildeman, 2016).

Unmet mental health service needs among children in out-of-home placements reflect serious missed opportunities to coordinate care and improve children’s well-being while they are in system custody. Formal collaborative partnerships between child welfare agencies and mental health providers can help foster linkages to services although front-line child welfare workers might need additional support to actualize these agency-level partnerships given the collaboration barriers they experience (Bai et al., 2009; Bunger et al., 2016; Fong et al., 2018; Hurlburt et al., 2004). For instance, child welfare workers might be untrained and unfamiliar with mental health issues (Dorsey et al., 2012), and find it difficult to prioritize children’s mental health service needs amid pressure to respond to safety concerns (Hoffman et al., 2016; Perez Jolles et al., 2019; Smith & Donovan, 2003). Even when child welfare workers identify children’s treatment needs, children can fail to receive care if workers are unfamiliar with treatment options (Bunger et al., 2009; Stiffman et al., 2000, 2004) or when there is limited availability of high quality, evidence-based treatment among providers who accept Medicaid (which covers services for children in out-of-home care) (Bruns et al., 2016; Scheeringa et al., 2020; Steinman et al., 2012). Attending to these barriers across both child welfare and mental health settings could potentially improve children’s mental health service access and their well-being.

### The Promise and Challenge of Implementing Service Cascade Models—The Gateway CALL Demonstration

Service cascades, similar to clinical pathways, are a type of cross-system intervention that link or integrate treatment services delivered in different systems to create a continuum of care from diagnosis to treatment (Belenko et al., 2017; Mugavero et al., 2013). When implemented at the intersection of child welfare and mental health, these interventions have potential to address the real-world barriers to identifying and connecting children to mental health treatment. Service cascades in this setting might include several sequenced components beginning with a screening and assessment in the child welfare system that lead to a referral and treatment in the mental health system (e.g. Barth et al., 2020).

Gateway CALL was a service cascade intervention designed and implemented within an urban county-based child welfare agency in a midwestern U.S. state that employs over 700 staff and serves 30,000 families annually. The agency designed Gateway CALL and implemented the intervention with children who entered child welfare custody and out-of-home placements to facilitate their access to mental health services and improve their mental health outcomes. The model included four components (screening, assessment, referral/linkage, and re-assessment) that address common challenges to identifying and connecting children to mental health services in typical child welfare practice. Gateway CALL was designed around an existing mental health assessment team (CALL clinicians) staffed by trained mental health clinicians from a local mental health provider. Having co-located mental health clinicians within the child welfare agency was a distinguishing feature of the intervention because it was intended to foster deeper integration of mental health expertise into service cascade components implemented within the child welfare agency and centralize coordination of mental health services for children.

#### Screening

The first component involved brief mental health and trauma screening conducted by intake workers in the child welfare agency to systematize identification of mental health service needs (instead of relying on worker discretion). In Gateway CALL, intake workers administered the Childhood Trust Events Survey (CTES; Pearl, 2000) to identify trauma exposure, and either the Devereaux Early Childhood Assessment (DECA; for children younger than six; LeBuffe & Naglieri, 1999) or the Strengths and Difficulties Questionnaire (SDQ, for children six or older; Goodman, 1997) to identify mental health disorder symptoms. Screenings were administered electronically on tablets with parents (and children who were 13 and older) during home visits at the time the case opened, and children were brought into child welfare custody. Children who scored above the threshold on either tool were electronically linked to a co-located CALL assessment team.

#### Assessment

The second component included case consultation and a comprehensive diagnostic assessment conducted by the co-located CALL team. CALL team clinicians consulted with the child welfare intake workers to learn about the family context for each child and share screening results. CALL clinicians then completed a diagnostic interview with the child and caregiver(s), obtained external records of past treatment history and had parents complete the Child Behavior Checklist (CBCL) for each child who was screened into Gateway CALL, and also administered the Youth Self Report (YSR) to youth aged 13 or older (Achenbach, 1991a, 1991b). Having a specialized mental health assessment team co-located within the child welfare agency was intended to expedite completion of a thorough diagnostic assessment (and intensive information gathering from parents) without children having to enter the mental health system, and foster information sharing and service coordination across systems.

#### Referral and Linkage

Third, CALL clinicians and ongoing child welfare workers (who assumed responsibility for coordinating child welfare service plans once cases were opened and transferred from intake) reviewed the results of the diagnostic assessment and made referrals/linkages to certified community-based mental health treatment providers who delivered high quality, specialty mental health services. As licensed and experienced mental health professionals, CALL clinicians had deep familiarity with local mental health providers, available evidence-based treatment modalities, and service quality to help drive referrals to appropriate treatment as indicated by assessment results. CALL clinicians also provided support to children’s caregivers to navigate the mental health system and link children to services. This is distinct from traditional child welfare practice where ongoing case workers refer children to services without the full information of a diagnostic assessment, robust understanding of treatment availability, consultation with a mental health clinician, or additional linkage supports.

#### Re-assessment and Case Monitoring

The fourth Gateway CALL component involved reassessments every 90 days while children remained in out-of-home care by the CALL team using the same CBCL and YSR assessments to monitor children’s progress in treatment. CALL clinicians shared results from the re-assessments with ongoing child welfare workers and provided consultation on treatment progress and child welfare case planning. (For additional intervention and implementation detail see Bunger et al., 2017, 2021).

Despite a deliberate intervention design that emphasized strong collaboration, responded to well-known barriers, and was designed to fit the local organizational context, earlier analyses suggested that Gateway CALL left many children with unmet mental health service needs. Although nearly all the children in the demonstration had some type of mental health diagnosis, fewer than half (47%) received treatment. Based on a quasi-experimental evaluation design (using a matched comparison group) Gateway CALL appeared to have no impact on children’s likelihood of receiving mental health services, although it might have increased the number of children’s mental health service visits (Bunger et al., 2021). In evaluations of similar types of demonstrations (that did not include the same type of intensive co-location), these types of cascades have demonstrated promise for quickly identifying children with extensive needs and recommending them for treatment (Akin et al., 2021; Verbist et al., 2020). Although a substantial number of children with service needs remained unserved in other demonstrations (Pullmann et al., 2018), those who received treatment in the community experienced symptom improvement (Bartlett et al., 2016, 2018). Taken together, while Gateway CALL and other similar types of models were designed to address barriers to identifying and linking children to mental health services, the effectiveness of these interventions remains unclear, though promising.

### Implementation Challenges Can Limit Fidelity and Cascade Effectiveness

Fidelity refers to the degree to which an intervention is delivered as intended (Carroll et al., 2007; Proctor et al., 2011). Poor implementation fidelity can limit the effectiveness of promising interventions when they are translated into real-world settings (Dusenbury et al., 2003). Fidelity reflects implementation quality or adherence to the content or core components of an intervention (Carroll et al., 2007). However, fidelity can be difficult to achieve especially for complex interventions like service cascades because they involve multiple components (e.g. screening, assessment, referral), implemented in multiple organizational or system environments (e.g. child welfare and mental health) (Dusenbury et al., 2003; Seys et al., 2019). Emerging literature highlights some of the challenges implementing and aligning service cascades (Belenko et al., 2017; Juckett et al., 2020; Van Deinse et al., 2019).

Because these cascades involve sequencing practice components across multiple organizations, difficulty implementing with fidelity at any stage or organization can compromise the effectiveness of the service cascade for improving clients’ service access and outcomes. The effectiveness of Gateway CALL and other similar cascades might have been limited because of poor implementation fidelity. Difficulty implementing the screening, assessment, referral, or case plan monitoring components could reduce children’s likelihood of receiving mental health services, or enough mental health service visits to lead to meaningful improvements in their outcomes (such as a reduction in behavior problems or mental health symptoms).

Understanding where model fidelity breaks down (in the cascade sequence or setting) can inform how system leaders select and target strategies for implementing these complex models. However, the implementation of these models and implications for service access and outcomes has received limited empirical attention. This manuscript draws on the Gateway CALL project as a case study to (1) assess fidelity to each component of the Gateway CALL cascade, (2) examine change in children’s mental health outcomes (specifically, their behavior problems) over time, and (3) evaluate the role of mental health service receipt on children’s mental health outcomes.

## Methods

### Study Design

Gateway CALL was rolled out in two waves across eight child welfare intake units responsible for investigating screened-in reports of child maltreatment (selected by agency leadership) beginning in February 2015 through July 2016. The larger study used a quasi-experimental design to examine whether Gateway CALL improved mental health service receipt, safety, and permanence outcomes for children in out-of-home care (Bunger et al., 2021). To address the aims of this manuscript, we draw on the longitudinal data from the experimental group only, which followed children in the study through January 31, 2017. Procedures were reviewed and approved by the IRB at the lead author’s institution.

### Participants

Participants included 175 children (from birth to age 18) who entered child welfare custody through one of the 8 experimental intake units between February 1, 2015 and July 30, 2016 and were placed in out-of-home care (e.g. foster care). Children were excluded if they were entering custody due to an event on an open case, were assigned to a managed care provider, or were in custody for fewer than two days (i.e., temporary emergency custody).

### Data Sources and Variables

We linked three administrative data sources. First, we drew on child welfare case records from the Statewide Automated Child Welfare Information System (SACWIS) to track all eligible children’s pathways through the child welfare system and basic case information. Second, we linked these records to children’s screening, assessment and re-assessment reports generated as part of this project and maintained separately at the child welfare agency in paper or electronic format. These records contained screening and assessment dates, responses to individual screening and assessment items, and aggregated scores. Finally, we linked children’s child welfare case records, screening results, and assessment reports with Medicaid billing records that reflected mental health services delivered to each child. These linked records were used to assess fidelity and children’s outcomes.

#### Fidelity

Fidelity was measured using four indicators that correspond with the four key stages of the intervention. *Screening fidelity* was operationalized as the percentage of Gateway CALL eligible children who received a mental health screening based on screening records linked with SACWIS data. *Assessment fidelity* was operationalized as the percentage of Gateway CALL eligible children who scored above the screening threshold and received an initial mental health assessment based on assessment reports. *Service fidelity* was operationalized as the percentage of Gateway CALL eligible children who scored above the screening threshold, had an initial assessment, and received specialty mental health treatment during the study observation period (between the time of mental health screening and January 31, 2017) as reflected in Medicaid billing records. To most closely capture treatment delivered by the children’s mental health system, specialty mental health treatment was defined as any service visit billed by a provider who was certified by the state Medicaid program as a mental health professional (e.g., psychiatrist, psychologist, or social worker). *Reassessment fidelity* was operationalized as the percentage of children who scored above the screening threshold, had an initial assessment, and at least one follow-up re-assessment approximately 90 days afterwards as reflected in the assessment and re-assessment reports. Higher percentages of children who received each phase of the intervention reflect stronger fidelity (with a goal of reaching 100%).

#### Mental Health Outcomes (Behavior Problems)

We examined children’s mental health outcomes based on both caregiver and youth reports of their behavior problems. Caregiver reports were assessed for children who received the Gateway CALL intervention (experimental group only) using the developmentally appropriate form of the CBCL (Achenbach, 1991a). The CBCL is a standardized caregiver-report measure that includes 113 items about children’s emotional and behavior problems. Caregivers rated their child on a 3-pont response scale (0 = not true, 1 = somewhat or sometimes true, 2 = very true or often true). Internalizing behavior problems (i.e., social withdrawal, somatic complaints, and anxiety/depression) were measured using the internalizing subscale and externalizing behavior problems (i.e., delinquency and aggressive behavior) were measured using the externalizing subscale. The total behavior problems were calculated by summing the internalizing and externalizing scores. The gender- and age-standardized T scores were used, with higher scores indicating greater symptoms. The CBCL was administered to caregivers by CALL assessment team members within 10 days of their child entering custody. The CBCL was re-administered with the primary caregiver within the child’s current placement every 90 days thereafter for the duration of the custody episode or until the end of the study observation period.

Youth ages 11–18 who received the Gateway CALL intervention also completed the YSR, a standardized, child self-report measure that is identical to the CBCL in content and structure (e.g., response categories) (Achenbach, 1991b). The YSR consists of 112 items that assess emotional and behavioral problems in the past 6 months. The same procedures described in the above (CBCL) were used to create internalizing, externalizing, and total behavior problem scores, at the same time intervals (upon entering custody and every 90 days afterwards). For both CBCL and YSR, T scores less than 60 are considered in the normal range, 60–63 represent borderline scores, and scores greater than 63 are in the clinical range.

#### Demographics

We extracted several child and family demographic features from SACWIS including age (in years as of January 31, 2017), sex (male or female). Children’s *race and ethnicity* was assessed categorically and reflecting major regional demographic groups (Black, white, or other). We also extracted information to understand other factors that might also drive children’s mental health service needs including the number of prior traditional (non-alternative response) screened-in reports of child abuse or neglect (*prior CAN*). From the most recent safety and risk assessment, we also extracted information about whether a child had special medical and behavioral needs, or a history of delinquency; or whether caregivers had substance misuse or domestic violence concerns (all dichotomous indicators where 1 = yes).

### Analysis

To understand fidelity to the Gateway CALL model for Aim 1, we used frequency analysis to examine the percentage of children (out of the 175 children in the intervention) who received each model component. To understand change in mental health outcomes (behavior problems) over time for Aim 2, we examined descriptively the percentage of children who scored above the clinical threshold (T score > 63) at initial and last assessment for each scale and based on both parent and youth report. We also used paired-samples t-tests to compare baseline behavior problems scores (the CBCL and YSR internalizing, externalizing, and total T scores) to scores on the final assessment. For Aim 3, we used linear mixed models to examine the relationship between mental health service receipt and change in symptom scores (internalizing, externalizing, and total) as reported by both parents and youth. Data were managed and analyzed using Stata (StataCorp, 2017) and SPSS v. 27 (IBM Corp, 2020).

## Results

### Sample Characteristics

Table 1 includes the demographic information of the sample. Children who participated in Gateway CALL were an average of 13.1 years old (SD = 5.4) and a slight majority were female (53.4%), 44% were Black, 35.6% were white, and 20.4% were some other race/ethnicity, including Asian, Hispanic, or multi-racial. On average, children had experienced an average of 2.9 prior child abuse or neglect reports (SD = 2.9), and 35.4% had a special need or history of delinquency indicated in their records. Over a third of children (37.1%) had lived in a home with caregiver domestic violence and 15.4% had caregiver substance use disorders indicated in their records.

**Table 1:** Gateway CALL sample characteristics (n = 175)

	%/M (SD)
Age	13.1 (5.4)
Prior CAN reports	2.9 (2.9)
Female	53.4%
Race/ethnicity	
Black	44.0%
White	35.6%
Other	20.4%
Special needs or history of delinquency	35.4%
Caregiver SUD	15.4%
Caregiver DV	37.1%

### Aim 1:  Fidelity to Gateway CALL

**Fig. 1:**
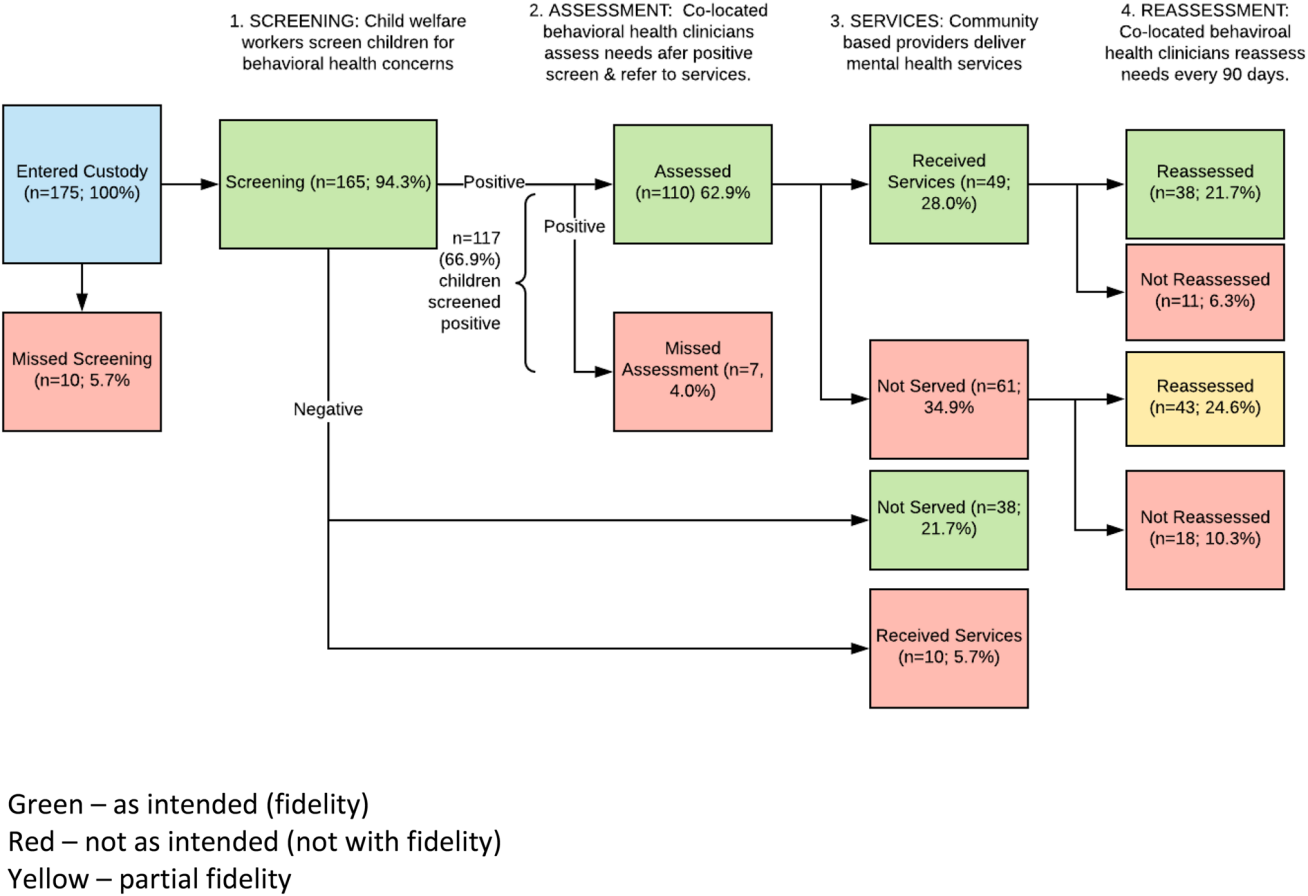
Children’s pathways through gateway CALL (n = 175 children)

**Fig. 2:**
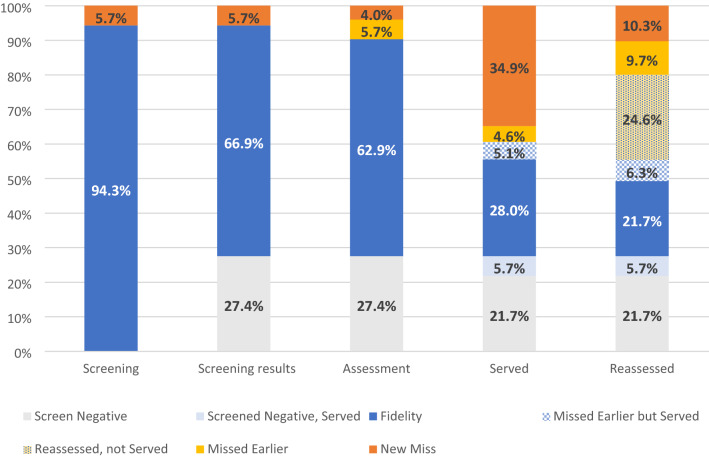
Fidelity to gateway CALL by component (n = 175 children)

Fidelity to the screening, assessment, service receipt, and reassessment/case monitoring phases of the Gateway CALL intervention are illustrated in Figs. 1 and 2; Table 2.

**Table 2:** Children who received each intervention component, and with fidelity (n = 175)

	All GWC children		GWC children with fidelity	
	n	%	n	%
Entered custody	175	100	175	100
Screening	165	94.3	165	94.3
Screened positive	117	66.9	117	66.9
Assessed	110	62.9	110	62.9
Served	68	38.9	49	28.0
Reassessed	81	46.3	38	21.7

#### Screening Fidelity

Of the 175 children entering the Gateway CALL units during the observation period, 165 (94.3%) were screened, and only 10 children (5.7%) were missed suggesting strong screening fidelity. A total of 117 children (66.9% of the 175 children in Gateway CALL, and 70.9% of the 165 screened) scored above the threshold on either the trauma exposure or mental health symptoms screening tools indicating a need for additional mental health assessment and services. At this stage, 48 children of the 175 children entering Gateway CALL (27.4%) scored below the threshold on both screening tools.

#### Assessment Fidelity

Children who screened positive on either screening tool were intended to be linked to the co-located CALL assessment team. Of the 175 children entering Gateway CALL, 110 (62.9%) received an initial mental health assessment, with either a completed parent or youth self-report. This number represents 94.0% of the children who scored above the screening thresholds indicating strong assessment fidelity. Only seven children (4.0%) were not assessed at this phase, in addition to the 10 children (5.7%) missed during the screening phase.

#### Service Fidelity

According to the model, we anticipated that most children who were assessed would be referred to specialty mental health treatment services, and because children in out-of-home placements are Medicaid-eligible, these treatment services should be captured in Medicaid billing records. However, only 49 children who received an assessment had any record of receiving a mental health visit in the Medicaid billing records suggesting poor service fidelity. These children who received mental health care after screening positive and receiving an assessment reflect only 28% of the 175 children in Gateway CALL. Records also suggest that some of the children who were missed in earlier screening and assessment phases (n = 9) or who screened negative (n = 10) also went on to receive mental health services, resulting in a total of 68 children (or 38.9% of the 175 children in Gateway CALL) who received services. A greater percentage of children who screened positive, and received an assessment (n = 61, 55.5% of those assessed and 34.9% of those in Gateway CALL) had no record of receiving mental health services in Medicaid billing records, reflecting unmet service needs.

#### Re-assessment Fidelity

In the final stage of Gateway CALL, all children who received an initial mental health assessment were to be reassessed every 90 days until the end of the demonstration or their stay in child welfare custody. A total of 81 children were reassessed (at least one record of a parent or youth self-report measure of behavior problems). These children account for only 46.3% of all 175 children in Gateway CALL, but 77% of the 110 children who received an initial mental health assessment suggesting that fidelity to the reassessment component was fairly strong. Most of those who were not reassessed (83%) left child welfare custody before the first 90-day reassessment would have been completed. The number of follow-up reassessments ranged from two to seven (Table 3). Of the children reassessed, 38 received mental health treatment as reflected in billing records; these children accounted for only 21.7% of all children in Gateway CALL. A slight majority of the 81 children who were reassessed (n = 43, 53%) did not receive treatment (according to billing records).

**Table 3:** Number of re-assessments at each 90-day interval

	Parent-report	Youth self-report
Baseline	110	97
Time 2	79	68
Time 3	60	51
Time 4	31	31
Time 5	23	21
Time 6	9	9
Time 7	2	4

### Aim 2: Change in Mental Health Outcomes (Behavior Problems)

**Fig. 3:**
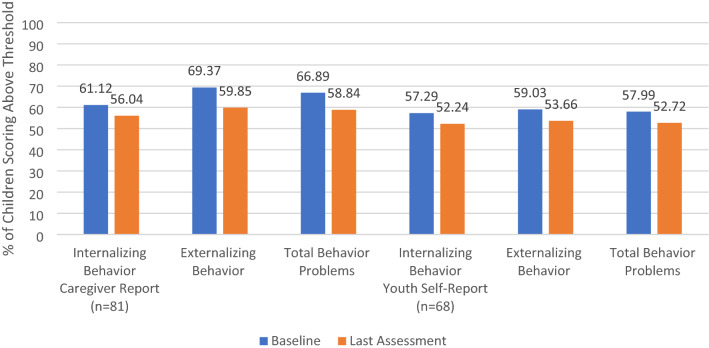
Change in percent of children scoring above the clinical threshold over time

Of the 81 children with at least one re-assessment, all 81 had at least-one parent reported re-assessment score, and 68 had at least one youth self-reported re-assessment score (Table 3). Behavior problems and their severity declined over time according to both parents and youth (Table 4; Fig. 3). Based on parent reports, the average total CBCL T-scores declined significantly from a baseline average of 66.89 (SD = 12.00) to a final average of 58.84 (SD = 10.97) [*t*(80) = 5.708, *p* < .001] which falls below the threshold for clinically significant behavior problems (T-score > 63). Similarly significant declines in parent reported internalizing [*t*(80) = 3.735, *p* < .001] and externalizing behavior problems [*t*(80) = 6.839, *p* < .001] were also observed. The percentage of children scoring above the clinically significant threshold for total parent-reported behavior problems declined from 75.3% at baseline to 39.5% at final reassessment, and similar decreases were observed for internalizing and externalizing behavior problems.

**Table 4:** Changes in behavior problems over time

	Parent report (n = 81)			Youth Self Report (n = 68)		
	Baseline	Final	t-test	Baseline	Final	t-test
Internalizing	61.12 (12.32)	56.04 (10.20)	t(80) = 3.490**	57.29 (13.60)	52.24 (13.08)	t(67) = 3.668**
Externalizing	69.37 (12.91)	59.85 (10.77)	t(80) = 6.502**	59.03 (11.93)	53.66 (11.20)	t(67) = 3.806**
Total	66.89 (12.00)	58.84 (10.97)	t(80) = 5.708**	57.99 (13.19)	52.72 (11.97)	t(67) = 3.935**

**p < .001

Changes in youth self-report of behavior problems were similar to those in the parent reports. Average total youth self-report T-scores declined significant from a baseline average of 57.99 (SD = 13.19), which is below the clinical cutoff, to a final average of 52.72 (SD = 11.97) [*t*(67) = 3.935, *p* < .001]. T-scores on youth self-reported internalizing [*t*(67) = 3.668, *p* < .001] and externalizing behavior problems [*t*(67) = 3.806, *p* < .001] also declined significantly. The percentage of children who scored above the clinically significant threshold on youth self-reported total behavior problems declined from 36.8% at baseline to 22.1% at the final reassessment.

### Aim 3: Mental Health Service Receipt and Mental Health Outcomes (Behavior Problems)

Table 5 summarizes the results from linear mixed models examining factors related to changes in parent reported behavior problems. Results suggest that the more mental health service visits a child received (in Medicaid billing records), the greater the decrease in parent reported internalizing (*b *= − .02, SE = .01, *p* = .019) and total behavior problems (*b* = − .02, SE = .01, *p* = .041). Children’s age was positively associated with parent reported internalizing behavior problems (*b* = .49, SE = .23, *p* = .038) suggesting that internalizing behaviors increased over time for older children. Child race, sex, maltreatment history, and whether or not children received any mental health services (as reflected in billing records) were not statistically significantly associated with changes in parent reported externalizing behavior problems.

**Table 5:** Linear mixed modelling results—changes in parent reported children’s behavior problems (n = 110)

	Internalizing			Externalizing			Total		
	B	SE	*p*	B	SE	*p*	B	SE	*p*
Any MH svcs (yes)	2.53	1.62	.120	2.18	2.05	.291	1.75	1.80	.332
No. of MH svcs	**− .02**	**.01**	**.019**	− .01	.01	.194	**− .02**	**.01**	**.041**
*Child race*									
Black	− 2.18	1.63	.181	1.82	2.06	.379	− .19	1.80	.917
Other	− .86	1.91	.654	− 1.35	2.46	.584	− 1.74	2.14	.420
Child sex (male)	− 1.37	1.44	.342	1.03	1.84	.576	− .38	1.61	.814
Child age (in years)	**.49**	**.23**	**.038**	.10	.30	.742	.28	.26	.290
Prior CAN	− .20	.22	.379	.16	.29	.579	.11	.25	.662

Bold values indicate *p* < .05

Table 6 presents the results from linear mixed models examining factors related to changes in youth self-reported behavior problems. Child age was negatively associated with changes in youth self-reported externalizing (*b *= − 1.75, SE = .64, *p* = .007) and total behavior problems (*b *= − 1.56, SE = .73, *p* = .035), suggesting that younger children reported greater decreases in their externalizing and overall behavior problems over time. Child race, sex, maltreatment history, and mental health service receipt (as reflected in Medicaid billing records) was not significantly associated with changes in youth self-reported behavior problems.

**Table 6:** Linear mixed modelling results—changes in youth reported behavior problems (n = 97)

	Internalizing			Externalizing			Total		
	B	SE	*p*	B	SE	*p*	B	SE	*p*
Any MH svcs (yes)	5.10	2.75	0.068	3.96	2.16	0.071	3.94	2.49	0.118
No. of MH svcs	− 0.02	0.01	0.102	− 0.01	0.01	0.414	− 0.02	0.01	0.135
Child race									
Black	− 5.45	2.79	0.054	− 2.85	2.19	0.197	− 4.83	2.52	0.060
Other	− 3.33	3.57	0.354	− 3.63	2.76	0.193	− 4.19	3.21	0.196
Child sex (male)	− 2.55	2.63	0.335	3.71	2.06	0.076	− 0.23	2.38	0.924
Child age (in years)	− 0.76	0.80	0.345	**− 1.75**	**0.64**	**0.007**	**− 1.56**	**0.73**	**0.035**
Prior CAN	− 0.50	0.45	0.268	− 0.16	0.34	0.654	− 0.14	0.40	0.725

Bold values indicate *p* < .05

## Discussion

Service cascades that link clients in one system (e.g. child welfare) to services in another (e.g. mental health) have potential to improve service access and client well-being although implementation challenges might compromise their effectiveness. In this study, we examined the implementation and child mental health outcomes of Gateway CALL, a system demonstration designed to link children in out-of-home placements to mental health care by implementing a sequence of mental health screening, assessment, referral, and case monitoring practice components within a child welfare agency. In earlier phases of the cascade (e.g. screening and assessment) where a mental health partner was well-integrated within the child welfare agency and practice, fidelity was strong. However, we found that implementation fidelity was poor for the later components (service receipt and reassessment) leaving many children with unmet mental health service needs. Despite these implementation breakdowns, children’s behavior problems improved over time; as children received more mental health service visits their parent-reported behavioral problems appeared to improve significantly. These results suggest that with special attention to implementation fidelity (especially at the point at which children are linked to the mental health system in the community) service cascade models have even greater potential for impact.

### Gateway CALL Fidelity

Implementation fidelity varied across Gateway CALL phases. Fidelity was strong across the initial screening and assessment cascade components implemented within the child welfare agency. It was clear that the child welfare intake workers and co-located CALL team clinicians successfully carried out the screenings and assessments together since there were few children missed during these phases. These results might reflect strong coordination between child welfare intake workers and CALL team clinicians, perhaps because of the co-location arrangement. However, fidelity dropped at the point at which children should have been referred to and received treatment in the mental health system. Only 28% of the children in Gateway CALL received at least one mental health service visit (with a certified mental health professional), even though over 63% had demonstrated need and received a full assessment. It is possible that children received supportive services (e.g., support groups, individual sessions) from outside of the mental health system. Besides the mental health system, children might often receive services in schools (Duong, et al., 2021). However, Gateway CALL occurred at a time when evidence-based mental health was limited in schools. It also may be that children received supportive services at other community-based organizations from professionals who were not certified mental health professionals (which would not be reflected in the Medicaid claims).

Although most children remained in child welfare custody for at least 90 days and were re-assessed by the CALL team at least once afterwards (where case workers and CALL team members may have followed up on missed service linkages), children still failed to receive specialty services. This suggests that later phases of the service cascade were not fully implemented and offers explanation for why children in Gateway CALL were no more likely to receive mental health services than children in a matched comparison group (Bunger et al., 2021).

Fidelity to the Gateway CALL model broke down at the point when children should have been referred and transitioned into community-based mental health services. Based on available data, it is difficult to pinpoint the problem—it is unclear whether children failed to receive specialized mental health services due to child welfare workers’ unfamiliarity or difficulty making referrals to certified providers (e.g. Bunger et al., 2009), limited mental health treatment availability and long waitlists for care (e.g. Barnett et al., 2018; Scheeringa et al., 2020; Steinman et al., 2012), or the challenges foster parents experience in bringing children to appointments (because the child refused, the provider was too far, scheduling, concerns about appropriateness, etc.) (Cao et al., 2019; Pasztor et al., 2006).

There were several collaboration breakdowns between the child welfare agency and its external partners that might explain why implementation suffered. First, there was a disruption in the contract for the co-located CALL assessment team leading to a change in provider halfway through the observation period. Contracting challenges in child welfare are common (e.g. Willging et al., 2015) and in our study, this provider change disrupted referral relationships which might have compromised implementation of the referral, treatment access, and re-assessment components. Second, limited collaboration with private foster care placement providers could have contributed to the drop off in mental health service receipt. In this agency, contracted placement providers (private non- and for-profit organizations) were responsible for placing children in foster care and arranging services in accordance with the case plan. Many of these providers preferred to conduct their own assessments and deliver in-house support services (perhaps for liability or billing reasons). As a result, these providers may not have accepted or supported the CALL team’s recommendations for specialized mental health services at other providers. Notably, while children might have received services delivered by contracted placement providers, unless they were delivered by a certified mental health professional and billed to Medicaid, they would not be considered specialty mental health services.

There were also issues within the child welfare agency that could have contributed to poor fidelity. High turnover rates among front-line staff, supervisors, and administrators could have undermined consistent follow-up with children and collaboration with mental health providers. Turnover can also contribute to institutional knowledge loss, decreased stakeholder buy-in, collaboration deterioration, and delays for necessary reorientation and partnership rebuilding (Gopalan et al., 2020; Whitaker et al., 2020) which affects implementation (Aarons et al., 2011; Rollins et al., 2010) particularly for service cascades (and other cross-system) interventions (Gopalan et al., 2021). Additionally, the intervention’s timing could have been problematic since screening occurred around the time children were removed from their home. This is a volatile time in a case, making it challenging to connect children to services; initiating the service cascade sooner (in the lifecycle of a family’s involvement in child welfare) might have led to better fidelity.

Given how service cascades are a series of interdependent steps, difficulty implementing even one component of the model can lead to overall implementation failures as we observed. These breakdowns might have been linked to difficulty collaborating effectively with external partners. The collaboration and implementation issues we experienced were not unique. Other demonstration sites also encountered significant challenges related to establishing strong collaboration across child welfare and children’s mental health systems (Akin et al., 2019; Barnett et al., 2016, 2018; Lang et al., 2017; Tullberg et al., 2017). It can take years to build capacity for working across systems (Connell et al., 2019), if at all (Jankowski et al., 2019) and these gains in collaboration can be difficult to maintain over time (Winters et al., 2020). Together the insights from Gateway CALL and other similar demonstrations suggest that effective collaboration strategies (e.g., co-locating staff, contracts with providers for expedited service access, clearly operationalized referral procedures) are likely essential for implementation success. Additional research on collaboration strategies could clarify how child welfare (and other human service systems) partner effectively with behavioral health organizations to implement cross-system models (Bunger et al., 2020; Hurlburt et al., 2004). This may be especially useful to child welfare systems partnering with behavioral health and other human service providers to scale up evidence-based parenting, mental health, and substance use treatment programs in communities as part of the Families First Prevention Services Act.

### Improvements in Mental Health Outcomes (Behavior Problems)

Despite poor implementation fidelity to the service receipt phase of Gateway CALL, children’s behavior problems and their severity declined over time. Notably, average final behavior problems scores fell below the threshold for clinically significant behavior problem. This suggests that children’s mental health improved. While the severity of children’s behavior problems might improve naturally over time once their living situations have stabilized (Rubin et al., 2007), the results of our linear mixed models suggest that children’s behavior problems improved more with greater numbers of mental health service visits. Our study design does not allow us to make inferences about whether service visits caused these improvements, although our earlier study findings suggest that the Gateway CALL intervention was effective for increasing the number of mental health service visits (Bunger et al., 2021). Attending more mental health service visits denotes stronger treatment retention, which is likely necessary for delivering a full dose of evidence-based interventions, and has been linked to better functioning outcomes for children (Foster, 2000). Thus, our results suggest that interventions like Gateway CALL have potential for improving children’s mental health by increasing the number of service visits they receive—if implemented with fidelity (where more children accessed mental health services), these models have real potential for broad impact for children in the child welfare system.

### Limitations and Future Directions

There are several methodological limitations that warrant consideration when interpreting our results. First, it is important to note that the measure of fidelity used during the study reflected model adherence only; our measure did not capture other dimensions of fidelity to the Gateway CALL model (e.g., dosage, quality) (Carroll et al., 2007) or whether services children received in the community were evidence-based which might also explain mental health outcomes (Ahn et al., 2016). Second, we were unable to gather data on mental health outcomes within the larger study’s comparison group, which limited our ability to infer causal relationships between service receipt and outcomes. During initial intervention and evaluation design, there were concerns about the ethics of administering diagnostic assessments or other measures about mental health symptoms to children in this population without also intervening. Additional controlled studies are needed to determine whether improvements in reported symptom severity are a result of treatment or other factors. Third, generalizability of this study’s findings are limited to similar types of urban county-based agencies situated within robust mental health service systems. Future studies are needed to understand whether this model yields similar outcomes when implemented in rural settings with more limited service availability (Cummings et al., 2016).

Fourth, the re-assessments might not have generated reliable and valid depictures of change in children’s behavior problems over time. Although the CBCL is a gold standard measure, it is completed by parents; in Gateway CALL, parents (or primary caregiver) completed the initial assessment, but because children were placed in out of home care, foster parents completed the re-assessments. For older children, we addressed this limitation by asking them to complete the YSR. While re-assessments completed by foster parents still generated useful clinical information for practice, our results about change in behavior problems among young children especially might be limited by this issue.

Finally, evidence from this study could be limited due to the use of administrative data; in particular, our data sources did not include an accurate and reliable indicator of mental health service referrals. As a result, we could not determine if Gateway CALL model fidelity broke down because the caseworker (or CALL clinician) did not refer children for services, or because of other problems that foster parents or others encountered while trying to follow through on the referral. Robust integrated data systems that reflect critical practice components are essential for testing and evaluating cross-system interventions like Gateway CALL.

## Conclusion

Service cascade models like Gateway CALL have potential to address unmet mental health service needs for children and youth in out-of-home placements. However, implementation issues can compromise their benefits. Our study demonstrates how children’s behavior problems improved with greater receipt of mental health services, but model fidelity can break down at the point where children transition across system boundaries compromising their service linkages. Our results suggest that strong and effective cross-system collaborations are essential for implementing and expanding the benefits of service cascades and other cross-system interventions.

## Supplementary Information

Below is the link to the electronic supplementary material.

Supplementary material 1 (DOCX 31.6 kb)
